# *Scolytus stepheni* sp. n. - a new species of bark-beetle (Coleoptera, Curculionidae, Scolytinae) from Northern India with a key to Indian *Scolytus* Geoffroy, 1762 species

**DOI:** 10.3897/zookeys.56.524

**Published:** 2010-09-17

**Authors:** Michail Yu. Mandelshtam, Alexander V. Petrov

**Affiliations:** 1 Bolshoy prospect, building 76, apt. 53, St.Petersburg, 199026, Russia; 2 Department of Ecology and Forest Protection, Moscow State Forest University, Mytishchi-5, 141005 Moscow

**Keywords:** Scolytus, Scolytinae, Curculionidae, new species, India, Stephen Lane Wood

## Abstract

A new species of bark-beetle from Kashmir, Scolytus stepheni **sp. n.**, dedicated to the late Professor Emeritus Stephen Lane Wood, is described and figured. Key to Indian Scolytus Geoffroy, 1762 species is provided.

## Introduction

In the recently published monograph by Maiti and Saha (2009) only three species from the genus Scolytus, namely Scolytus major Stebbing, 1903, Scolytus kashmirensis Schedl, 1957 and Scolytus chelogaster Schedl, 1957 are mentioned as occurring in India. Schedl’s paper about Indian bark and timber beetles (1957) also lists Scolytus nitidus Schedl, 1936, Scolytus scolytus (Fabricius, 1775) from India and Scolytus rugulosus (Müller, 1818) var. baluchistani, n.var. and Scolytus amygdali Guérin, 1847 as Scolytines native to the Indian subcontinent. As we have demonstrated, Scolytus scolytus was wrongly recorded from India and in fact the records of this species refer to the newly described species Scolytus stepheni sp. n. Although Scolytus rugulosus and Scolytus amygdali were recorded from Pakistan, we found it desirable to include these species into the key to the Indian Scolytus species provided below.

The two specimens considered in the current paper were labeled by K. Schedl and cited by him (Schedl 1957) as Scolytus scolytus (Fabricius, 1775), despite clearly different frontal vestiture, and abdominal armature in the male. The species was mentioned by Beeson (1941) as Scolytus himal-ulmi but without any description (Schedl 1957). We have considered the status of the new species with Carolus Holzschuh who first labeled the species as new, and he agreed that the present authors should describe the new species. Scolytus scolytus should be excluded from the Indian Scolytinae fauna.

## Systematics

### Scolytus stepheni sp. n.

urn:lsid:zoobank.org:act:951F18E6-5F12-4AE2-B233-3AB0C1609E05

Figs 1 3, 4 

#### Type material.

Holotype (male) (NHMW) bears the following labels: 1.Pahlgam, 7000 ft, Lidar valley, Kashmir. 7.VI.1928 C.F.C. Beeson. (on reverse side t.№193) // 2. Male sign // 3. under bark Ulmus wallichiana // 96.

Allotype (female) (NHMW) bears the following labels: Pahlgam, 7000 ft, Lidar valley, Kashmir. 7.VI.1928 C.F.C. Beeson // Female sign // under bark Ulmus wallichiana // Scolytus scolytus Geoff. [Sic!] det. K.E.Schedl 1953 // n.sp. det. C. Holzschuh.

**Figure 1.**
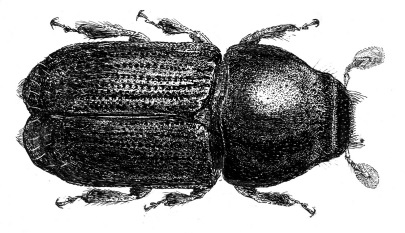
Scolytus stepheni, sp. n. male dorsal view.

**Figure 2.**
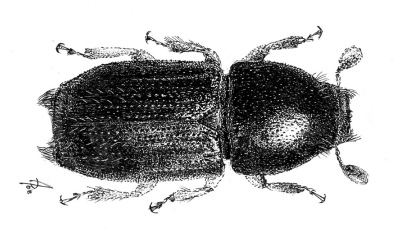
Scolytus dahuricus Chapuis, 1869 male dorsal view.

#### Description.

##### Holotype

###### Male.

Body length 4.6 mm, 2.1 times as long as wide

Head black, faintly shining. Frons flat, longitudinally aciculate, its surface with fine pale hair-like setae; lateral parts of frons near eyes covered by denser and longer hair-like setae. Vertex deeply punctured. Antennae brown, antennal club nearly elliptical, its surface with short golden hair-like setae.

Pronotum reddish-brown, nearly as long as wide, wider than its length. Sides of pronotum parallel for most of their length; gently rounded towards pronotal apex; constriction in apical portion of pronotum only weakly developed. Surface punctured, with punctures larger in frontal portion than in center of pronotal disk. Anterolateral angles of pronotum bear sparse long light hair-like setae.

Elytra reddish-brown, faintly shining, as wide as pronotal base and 1.5 times as long as pronotum. Elytral base slightly elevated; scutellum triangular; impression near scutellum only poorly developed. Striae slightly impressed, strial punctures circular, closely placed; interstriae flat with smaller punctures than in striae and less closely placed. Subapical elytral constriction distinct. Prior to apex (in subapical constricted part) elytra with faint impression with irregularly set punctures. Pale sparse erect hair-like setae only visible near elytral apex.

Abdomen reddish-brown, dull. First and second sternites darker than third, fourth and fifth sternites. All sternites with densely set round punctures. Posterior margins of third and fourth sternites with minute median tubercles. Lateral sides of fifth sternite clearly thickened on posterior margin and with carinate apex. Two strong tubercles developed on fifth sternite, their apices with brushes of densely set golden hair-like setae, the brushes not confluent at glabrous apical margin of abdomen.

Legs reddish-brown, covered by golden hair-like setae.

###### Female.

Similar to male but can be distinguished by the larger size (4.9 mm), more convex frons and by less strongly developed “callous-like elevations” at 5-th sternite without hair-like brushes of setae.

**Figures 3, 4.**
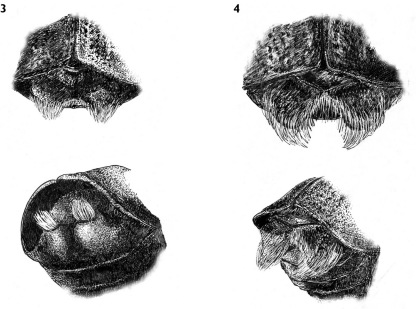
**3** Scolytus stepheni male, abdominal apex **4** Scolytus dahuricus male, abdominal apex.

#### Diagnosis.

The new species is more closely related to Scolytus dahuricus (Fig. 2) than to any other Indian Scolytus species. From Scolytus dahuricus it is distinguished by the broader body, by the flat elytral interstriae without any transverse rugosities or furrows. In the new species, the hair-like setae at the lateral parts of the frons are shorter and sparser. The clearest difference from Scolytus dahuricus is seen in the form of the tubercles on the fifth abdominal segment and in their vestiture. In Scolytus dahuricus long hair-like setae, forming tufts attain the apical margin of the fifth sternite where the two tufts are confluent, and intergrade. The tubercles of the fifth sternite in Scolytus dahuricus are spatuliform, blade-like whereas in Scolytus stepheni these tubercles have the form of truncated cones. The tubercles in Scolytus stepheni are set closer to the median line of the fifth sternite, whereas in Scolytus dahuricus the tubercles occupy the whole space from center to lateral sides of sternite. The distance from the tubercle apices to the posterior margin of the fifth sternite is significantly greater in Scolytus stepheni compared to the relevant distance in Scolytus dahuricus.

#### Etymology.

Professor Stephen Lane Wood worked extensively on collections of Scolytinae preserved in Indian museums, and described a number of new Scolytinae species from India. In this paper we describe one more new bark-beetle species from India kept in the Natural History Museum in Vienna (NHMW) and dedicate this species to the late Professor Stephen Lane Wood.

## Discussion

The new species is closely related to Scolytus dahuricus Chapuis, 1869, but differs in many details. Body length 4.6 mm, 2.1 times as long as wide (in Scolytus dahuricus body is 2.3–2.6 as long as wide). Other features to distinguish new species from Scolytus dahuricus are given in the section Description and Diagnosis. To help researchers to deal with Indian Scolytus species we add below the key to species that is absent in Maiti and Saha (2009) monograph.

## Key to the Indian Scolytus Geoffroy, 1762 species

**Table:** 

1.	Abdomen unarmed in both sexes, bearing no processes or tubercles on the second sternite or high-elevated callosities on the 5-th sternite, these callosities with or without brushes of hair-like setae	2
–	Abdomen is modified in both sexes, bearing either process or tubercle(s) on the second sternite, or highly elevated callosities on the 5-th sternite, these callosities with or without brushes of hair-like setae	4
2(1).	Punctures nearby anterior and lateral margins of the pronotum are elongate and significantly denser than on the pronotal disk; these punctures nearby anterior and lateral margins fuse with neighboring punctures, forming longitudinal rugosities. Pronotum in middle part with distinct, strong puncturation. Beetles small, usually 1.4–2.4 mm, rarely up to 3 mm in length	Scolytus rugulosus (Müller, 1818)
–	Pronotal punctures nearby anterior and lateral margins of the pronotum set denser than on the pronotal disk, but even at margins punctures are clearly divided from neighbors, never fuse and do not form longitudinal rugosities. Beetles usually of larger size	3
3(2).	Punctures in elytral striae and interstriae of approximately the same size. Pronotal disc puncturation consists of minute and sparse elongate punctures; in the middle of pronotum punctures are separated greater than by 5 times the width of an individual puncture as in European Scolytus mali. Both sexes with median frontal tubercle above epistoma. Frons is convex in both sexes, covered by granules, not aciculate even in the upper portion. Elytra unicolorous, dark brown, with the rows of erect interstrial hair-like setae evident from base of the elytra and up to their apex. Abdomen is ascending gradually, second sternite is convex. Body 2.8–6.0 mm in length. On Conifers	Scolytus major Stebbing, 1903
	(= Scolytus minor Stebbing, 1903; = Scolytus deodara Stebbing, 1903)	
–	Punctures in elytral striae evidently larger than punctures of interstriae. Pronotal disc puncturation consists of elongate punctures, not forming longitudinal rows; in the middle of pronotum punctures are separated by 2–3 times the width of an individual puncture essentially as in European Scolytus laevis. Frons longitudinally aciculate. Relatively pale elytra usually with a dark transverse band near middle. Beetles of smaller size, 2.0–3.0 mm in length. Mainly on Rosaceae.	Scolytus amygdali Guérin, 1847
4(1).	Fifth abdominal sternum with two adjacent conical processes, each process with a bundle of at least 15 golden hair-likesetae. These processes are set a bit apart from the posterior margin of the 5 th sternite. Body length 4.6 mm	Scolytus stepheni sp. n., male
–	Fifth abdominal sternum without conical processes bearing bundles of hair-likesetae, it may have at most highly elevated callosities separated by the longitudinal sulcus	5
5(4).	Fifth abdominal sternum with two callous-like elevations separated by the longitudinal impression. These callous-like elevations do not touch the posterior margin of the 5 th sternite. Frons uniformly punctured, evenly covered with short hairs that are far not so dense as in European Scolytus scolytus (Fabricius, 1775) and somehow longer. Body length 4.9 mm	Scolytus stepheni sp. n., female
–	Fifth sternite not modified, it may have only slight impression encircled by the elevated posterior margin of the sternite	6
6(5).	Tubercle of the second abdominal sternite is small, conical and sharp; this tubercle is set nearby the posterior margin of the second sternite, but not at the posterior margin itself. This tubercle is slightly larger in male than in female. Second abdominal sternite strongly ascending. Pronotum with small and widely separated punctures essentially as in European Scolytus mali. Scutellum and scutellar impression are covered by white elongate hair-like setae in inabraded specimens. Punctures of elytral striae are large, round and shallow, only slightly larger compared to interstrial punctures which are also round and shallow. Frons in male is flattened, aciculated, each side of the frons has a row of long golden hair-like setae sloping from vertex to epistoma, each seta exceeding half of the frontal width. In females frons is in rather dense long hair-like setae throughout the whole surface, the individual hair-like setae are less than 1/3 of the frontal width. Body length 2.9–3.4 mm	Scolytus nitidus Schedl, 1936
–	Second abdominal sternite with the tubercle or process in the anterior third of sternite length. Second abdominal sternite subvertical	7
7(6).	Posterior margin of the second abdominal sternite unmodified. Second abdominal sternite with the long process in the anterior third. In males this process is long, horizontal, suddenly and sharply curved upwards at its apex; in female the process is sword-like, more short and more gently curved upwards at is apex. Puncturation of pronotum resembles European Scolytus laevis and not Scolytus mali, pronotal points are distinct and rather large. Elytra are brown with the black central portion, forming an obscure dark band nearby middle. Scutellum is deeply set in scutellar impression, glabrous. Elytral interstriae each with one row of regularly set minute punctures, much smaller compared to points of striae. In male frons impressed in the middle, shining, with deep punctures and deeply, but not densely longitudinally aciculate. Hair-like setae of the upper portion of the frons form two brushes at the border with vertex; besides at the interior margin of each eye there is a bundle of enormously long hair-like setae in number of 9; these setae are incurved and nearly equal the width of the frons, at least exceeding 2/3 of its width. Female frontal vestiture is not preserved in available specimen. Body length 3.5 mm	Scolytus chelogaster Schedl, 1957
–	Posterior margin of the 2nd abdominal sternite is modified. In females it is carrying two sharp denticles set one fifth of sternite width apart from sternite lateral margin and in males there are two strong conical denticles set at the posterior sternite margin at one fouth of sternite width apart from it lateral margin. Central portion of second sternite posterior suture is thickened in females and in males this portion of sternite forms a triangular elevation projecting backwards and overhanging the third sternite. Besides, in males there is a button-like tubercle in the middle of the anterior third of second sternite; in females this tubercle is larger and is laterally compressed. Puncturation of the central part of the pronotum is minute, essentially as in Scolytus mali. Anterior 2/3 of pronotum is black, posterior part of the pronotum and elytra completely are light brown in colour. Scutellum set deeply in scutellar impression and is covered with elongated scale-like white setae. Elytral strial and interstrial punctures are large, round and shallow, nearly equal in size. Posterior portion of elytra, base of the second abdominal sternite, fifth abdominal sternite and hind legs are covered by the very long hair-like setae not forming tufts anywhere. Male frons at sides with two parallel rows of long setae, individual setae equal to one half of the frontal width; there are no bundles of long setae at interior margin of the eyes. In female frons is covered by sparse erect hair-like setae throughout all frontal surface. Body length 3.6 in the male and 4.1 mm in the female	Scolytus kashmirensis Schedl, 1957

## Supplementary Material

XML Treatment for Scolytus stepheni

## References

[B1] Beeson CFC (1941) The ecology and control of the forest insects of India and the neighbouring countries. The Author, Dehra Dun: 1007 pp.

[B2] Maiti PK Saha N (2009) Fauna of India and the Adjacent Countries : Scolytidae: Coleoptera (Bark-and Ambrosia-Beetles), Vol. I (Part 2). Zoological Survey of India, Kolkata, 246 pp.

[B3] Schedl KE (1957) Indian bark and timber beetles I. 160. Contribution to the morphology and taxonomy of the Scolytoidea. Indian Forest Records. 9(7): 165–169

